# Alveolar macrophages and airway hyperresponsiveness associated with respiratory syncytial virus infection

**DOI:** 10.3389/fimmu.2022.1012048

**Published:** 2022-10-20

**Authors:** Yuxin Wang, Junwen Zheng, Xia Wang, Pu Yang, Dongchi Zhao

**Affiliations:** ^1^ Department of Pediatrics, Zhongnan Hospital of Wuhan University, Wuhan, China; ^2^ Children’s Digital Health and Data Center of Wuhan University, Wuhan, China

**Keywords:** respiratory syncytial virus, alveolar macrophages, polarization, immune regulation, airway hyperresponsiveness

## Abstract

Respiratory syncytial virus (RSV) is a ubiquitous pathogen of viral bronchiolitis and pneumonia in children younger than 2 years of age, which is closely associated with recurrent wheezing and airway hyperresponsiveness (AHR). Alveolar macrophages (AMs) located on the surface of the alveoli cavity are the important innate immune barrier in the respiratory tract. AMs are recognized as recruited airspace macrophages (RecAMs) and resident airspace macrophages (RAMs) based on their origins and roaming traits. AMs are polarized in the case of RSV infection, forming two macrophage phenotypes termed as M1-like and M2-like macrophages. Both M1 macrophages and M2 macrophages are involved in the modulation of inflammatory responses, among which M1 macrophages are capable of pro-inflammatory responses and M2 macrophages are capable of anti-proinflammatory responses and repair damaged tissues in the acute and convalescent phases of RSV infection. Polarized AMs affect disease progression through the alteration of immune cell surface phenotypes as well as participate in the regulation of T lymphocyte differentiation and the type of inflammatory response, which are closely associated with long-term AHR. In recent years, some progress have been made in the regulatory mechanism of AM polarization caused by RSV infection, which participates in acute respiratory inflammatory response and mediating AHR in infants. Here we summarized the role of RSV-infection-mediated AM polarization associated with AHR in infants.

## Introduction

Respiratory syncytial virus (RSV) is the dominant cause of lower respiratory tract infection in children younger than 2 years of age worldwide. It is estimated that 4 million children are admitted to hospitals for RSV infection and 200,000 of the hospitalized children die each year (1, 2). Due to the immature composition and functions of their immune cells and molecules, infants infected with RSV often progress to lower respiratory tract inflammation, and some of them can develop a chronic lung disease (3, 4). When re-infected or exposed to allergens, this infection in infants can manifest as recurrent wheezing. The pandemic of the coronavirus disease 2019 (COVID-19) has changed the epidemic pattern of RSV; it is estimated that the recurrence of RSV will be more intense in the future and may become a major economic burden to society (3, 4).

Alveolar macrophages (AMs) are the important part of the respiratory tract’s innate immune barriers and play a key role in engulfing pathogens and antigen presentation (5, 6), and together with epithelial cells, contribute to setting the threshold and the quality of the innate immune response in the acute and convalescent phases of RSV infection. It has been reported that AM polarization is driven by RSV in a variety of microenvironments to exert multiple biological effects (7). Polarized AMs participate in local inflammatory responses and in mediating intercellular communication to stimulate naive lymphocyte differentiation (8, 9), thus regulating the intensity of the inflammatory response, which is associated with immunosensitization and the pathology of airway hyperresponsiveness (AHR) in the late life of infants infected with RSV (10–12). Therefore, immunomodulatory therapy targeting AMs may be one of the approaches to further explore effective treatment strategies. In this paper, we summarize the potential association between AM polarization and AHR after RSV infection in infants.

## RSV infection and host response

RSV is a single-stranded negative-sense RNA virus belonging to the Pneumovirus genus of the Paramyxoviridae family (13). Its genome can encode 11 proteins that play roles in mediating viral replication, packaging, and assisting the virus to escape immune surveillance. Glycoprotein binds to glycosaminoglycans on the cell surface, interfering in immune cell recruitment and various cytokine production. Fusion protein mediates the fusion between the virus and the cell membranes of the host to form syncytia. Non-structural protein 1 and 2 inhibit interferon (IFN) production and its signaling conduction (14). Phosphoprotein inhibits exogenous apoptotic signals and contributes to persistent RSV infection in macrophage-like cells (15). By disrupting the host gene transcription and interfering with the synthesis of mitochondrial proteins, matrix protein weakens the body’s immune recognition of RSV (16).

**Figure 1:**
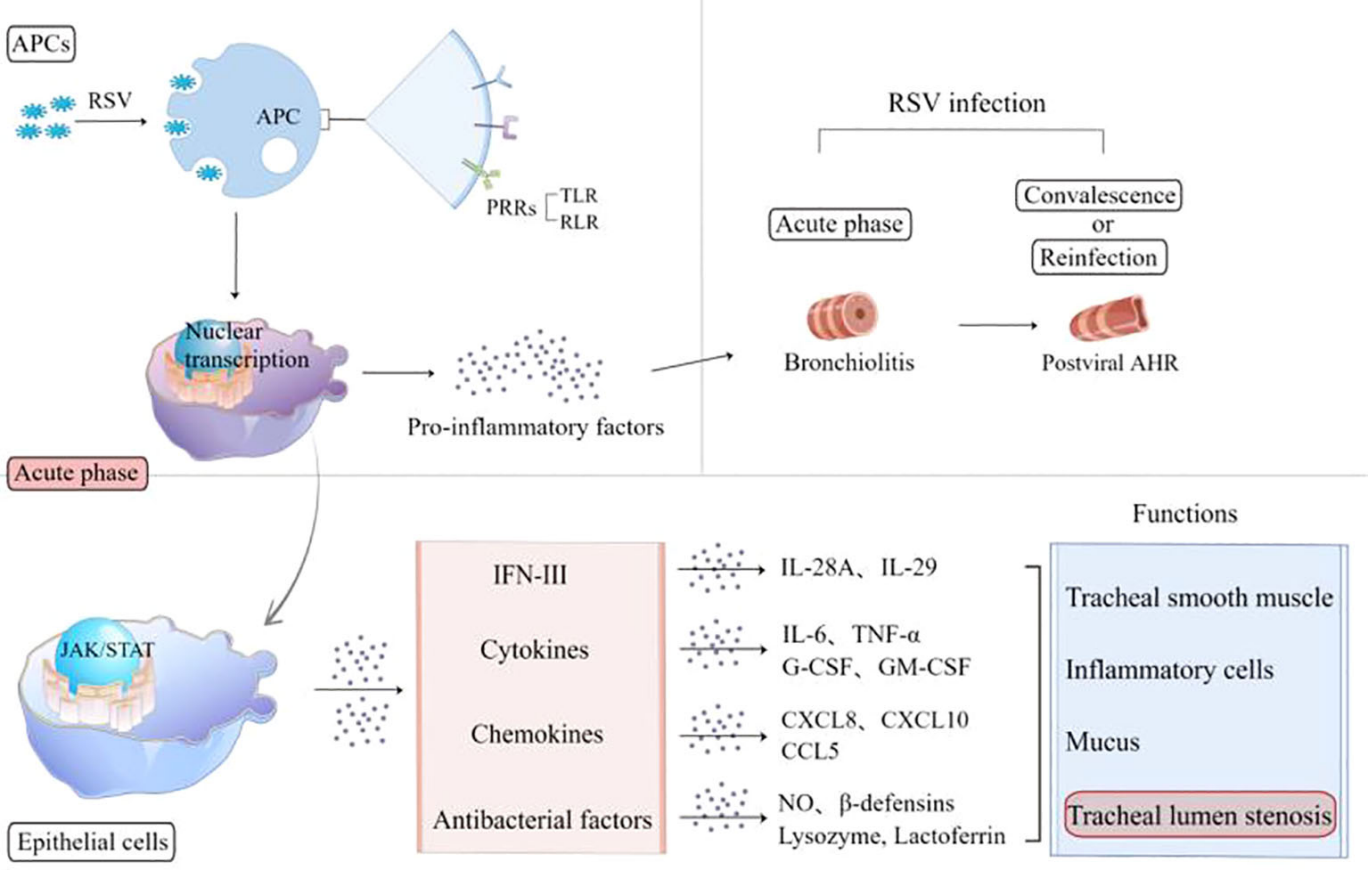
RSV activates APCs to express PRRs, including TLR and RLR, and release pro-inflammatory factors. In the acute phase, RSV-infected epithelia express interferons, cytokines, chemokines, and antimicrobial factors involved in the airway inflammation reaction. The pathological lesions caused by these inflammatory mediators experience two stages: bronchiolitis and post-viral AHR. APCs, antigen-presenting cells; RSV, respiratory syncytial virus; PRRs, pattern recognition receptors; TLR, Toll-like receptor; RLR, retinoic acid-inducible gene 1 (RIG-I)-like receptor; AHR, airway hyperresponsiveness; IFN, interferon; IL, interleukin; TNF-α, tumor necrosis factor-α; G-CSF, granulocyte colony-stimulating factor; GM-CSF, granulocyte–macrophage colony-stimulating factor; NO, nitric oxide.

In host cells, RSV activates pathogen-associated molecular patterns (PAMPs), which promotes the maturation of antigen-presenting cells (APCs) to express pattern recognition receptors, toll-like receptors (TLRs), and retinoic acid-inducible gene 1 (RIG-I)-like receptors (RLRs) (17–19). RSV can also invade lung macrophages directly, is recognized by mitochondrial antiviral signaling protein (MAVS)-coupled RLR (12), and can activate nuclear transcription to regulate innate immune responses (**Figure 1**). The expression of pro-inflammatory mediators and the recruitment of inflammatory cells to the infected or injured tissue and their migration across the endothelium are crucial events in early immune extravasation defense against RSV infection (20).

AM-mediated lung pathological lesions are usually not invaded by RSV directly, but mainly immune-mediated inflammatory responses (21). The acute infection phase is dominated by airway inflammation such as bronchiolitis, and the convalescent phase is characterized by airway hypersensitivity. Both of them belong to airway hyperresponsiveness. A variety of molecules are involved in the acute phase across epithelial cells (ECs), including interleukin (IL)-6, tumor necrosis factor-α (TNF-α), granulocyte-colony stimulating factor, granulocyte-macrophage colony stimulating factor (GM-CSF), chemokines (CXCL8, CXCL10, and CCL5), antibacterial factors including nitric oxide (NO), β-defensins, lysozyme, and lactoferrin (10, 17), which might cause tracheal smooth muscle spasm, hyperemia, edema, inflammatory cell aggregation, secretion, and cell shedding to block the airway (22–25). Reinfection or inhalation of allergens during the convalescent period can both trigger the overexpression of CD8 T and Th2-like cytokines involved in triggering wheezing.

## Classification and characteristics of AMs

**Figure 2:**
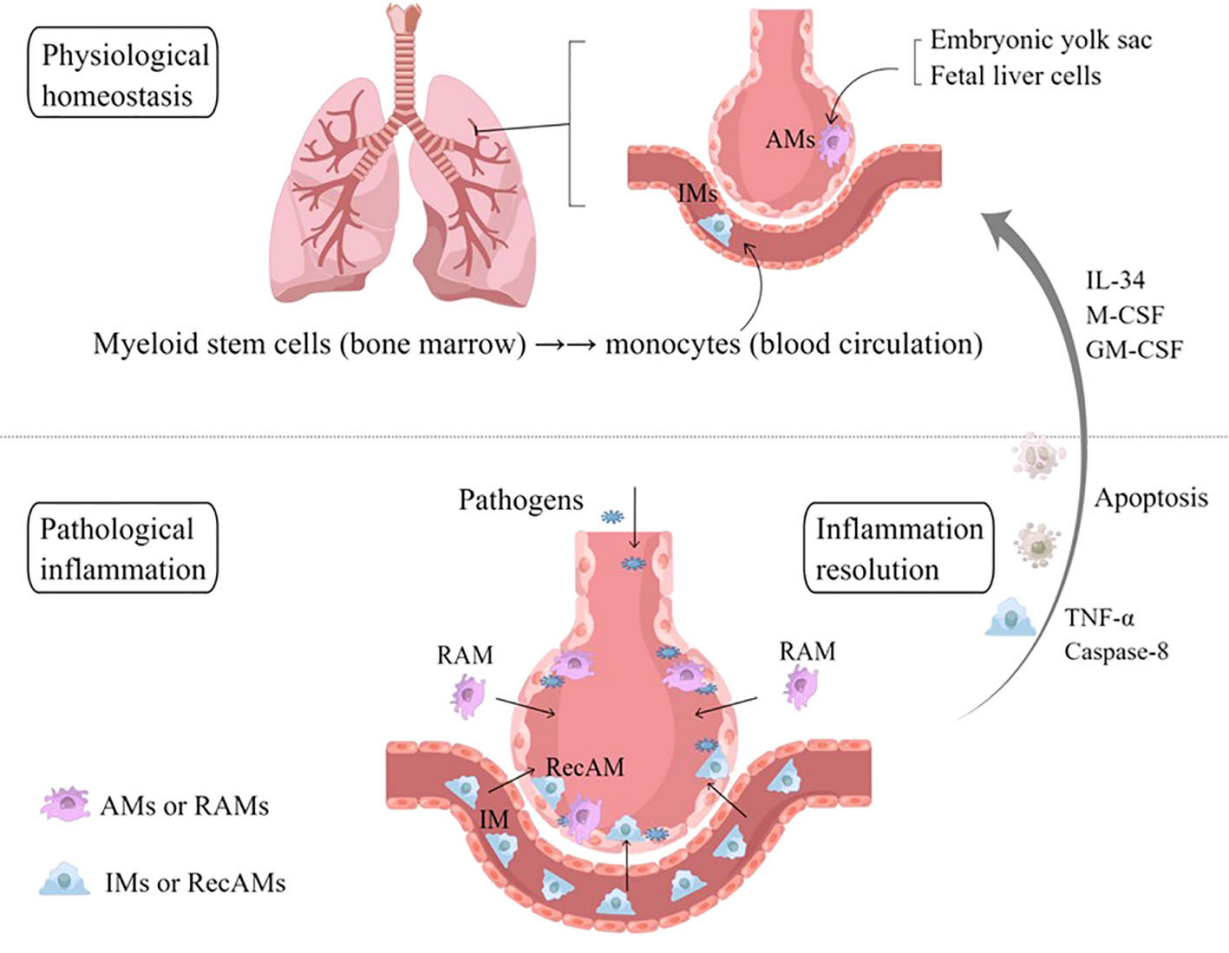
Sources and classification of AMs. In physiological homeostasis, AMs are equivalent to RAMs, which originate from embryonic yolk sacs and fetal liver cells. In the event of a large number of microbial invasion or inhalation of allergens, IMs are recruited into the alveoli, known as RecAMs. After the inflammation subsides, RecAMs coming from IMs will undergo programmed apoptosis, while RAMs maintain their original distribution characteristics under the action of IL-34, M-CSF, and GM-CSF. AMs, alveolar macrophages; IMs, interstitial macrophages; RAMs, resident airspace macrophages; RecAMs, recruited airspace macrophages; IL, interleukin; GM-CSF, granulocyte–macrophage colony-stimulating factor; M-CSF, macrophage colony-stimulating factor; TNF-α, tumor necrosis factor-α.

Lung macrophages are usually divided into two subpopulations depending on their distinct locations: AMs located on the surface of the alveoli cavity and interstitial macrophages (IMs) located in the interstitial pulmonary stromata (26, 27). In inflammatory states, AMs are recognized as the resident airspace macrophages (RAMs) and the recruited airspace macrophages (RecAMs), depending on their origins and wandering characteristics (**Figure 2**) (28, 29).

RAMs are steady-state “AMs” that derive mainly from embryonic yolk sacs and fetal liver cells (30), which reside on the surface of the alveoli cavity for a long time. RAMs are not evenly distributed in each alveolus, and notably only 30–40% of alveoli contain RAMs. Most of the RAMs crawl in and between alveoli through the pores of Kohn to monitor the microenvironment, while the remaining 10% of RAMs are entirely sessile (5). In the physiological environment, there is contact inhibition between RAMs, which contributes to preventing RAMs from accumulating in the alveoli. This distribution characteristics are regulated in part by IL-34 and macrophage-colony stimulating factor (M-CSF) in the alveoli (31). Through the regulation of GM-CSF and the mechanistic target of rapamycin complex 1, RAMs, as long-lived cells, can proliferate *in situ* to replenish themselves without the need for mononuclear macrophages from circulating blood as supplement or replacement, with an annual renewal rate of about 40% (32). GM-CSF have been confirmed to upregulate the expression of anti-apoptotic genes in RAMs, which is necessary to promote maturation and prolong their lifespan (5, 33).

RAMs, being capable of engulfing foreign particles and endogenous proteins (including surfactants and cell debris) to initiate an immune response, play a key role in regulating the innate immunity of the respiratory system and preventing infection from inhaled pathogens. Moreover, together with alveoli ECs, RAMs can also contribute to maintaining lung tissue homeostasis and the intensity of the inflammatory response (34). The distributions of RAMs in the steady-state microenvironment are in the dynamic equilibrium of “self-sufficiency”. During endotoxin-induced acute inflammation or exposure to a large number of pathogens, RAMs are the first sentinel of the respiratory tree and constitute the dominant immune cell in the steady state to metabolize pro-inflammatory effectors, including the recruitment of platelets, neutrophils, and other inflammatory cells, which contribute to co-participating in and regulating the onset and development of the disease (35).

RecAMs belong to the subpopulation of IMs that travel towards the site of inflammation in the alveolar cavity in pathological conditions. IMs originate in bone marrow monocytes, circulating through the bloodstream into the interstitial tissues of the lungs and being in transitional states. IMs can patrol in the interstitium of different alveoli, where they identify different inflammatory or necrotic and exfoliated cells and exert a phagocytic effect, which, in turn, release IL-10 to maintain microenvironment homeostasis (9, 36). In the acute phase of infection, IMs will be chemotactic to the alveoli cavity and recruited to become RecAMs (37). In addition, RNA gene sequencing showed that the immunoprogramming of RecAMs was dynamic (32, 35) and could develop into the same phenotype and provide the same functionality as RAMs during the peak inflammatory periods (38, 39), including the production of pro-inflammatory cytokines and elimination of pathogens. RecAMs release anti-inflammatory factors to repair pathologically damaged tissues when the inflammation is subsiding. RecAMs program apoptosis after the inflammation is gone, whereas RAMs will continue to survive and sustainably replenish themselves. This causes the amount of AMs to form an emergency dynamic cycle between the homeostasis phase and the inflammatory phase (32).

## Inflammation-activated AM polarization

Both RAMs and RecAMs can be activated to divide into M1 and M2 phenotypes according to the microenvironment changes (5). Conventional studies label nitric oxide synthase (NOS) and arginase (Arg) to determine the activation states of M1 and M2, respectively. However, recent studies have shown that both NOS and Arg can be co-expressed within the same cell (32), and AM polarization is not a distinct “dichotomy” but is multidimensional, dynamic, and complex (40). Moreover, the classic “M1 and M2” classification remains representative. M1-like macrophages exacerbate the airway inflammatory response that may be associated with long-term airway sensitization (41). In contrast, M2-like macrophages are capable of anti-inflammatory responses and repairing damaged tissues to maintain immunity balance (5). Once the microenvironment of the alveolars changes, the phenotypes and the functions of M1 and M2 could be reversed.

**Figure 3:**
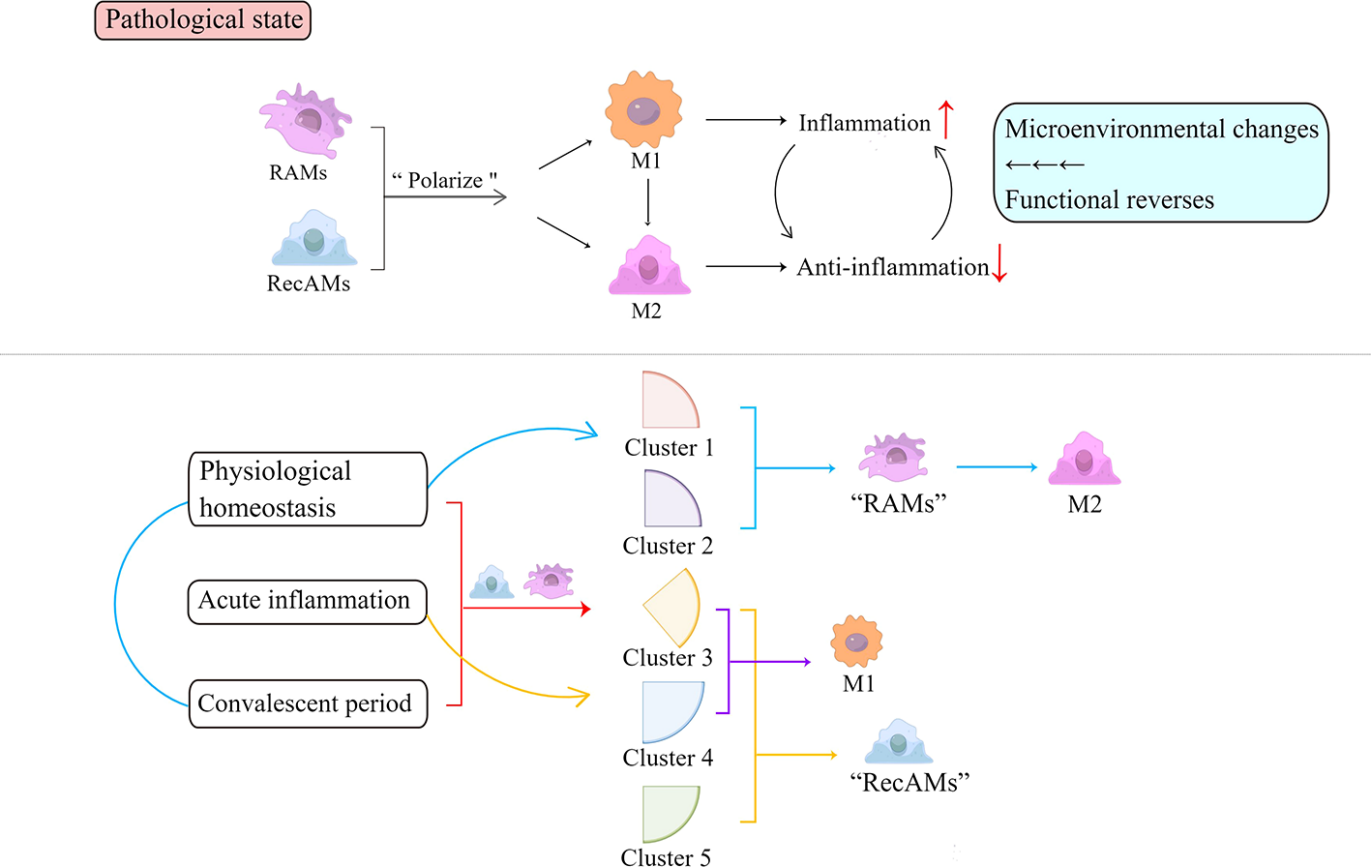
Inflammation activates the polarization of AMs. Depending on functions, polarized AMs are roughly divided into pro-inflammatory M1 and anti-inflammatory M2. Based on their single-cell RNA sequencing analysis, AMs can be identified as five clusters at three time points throughout the inflammatory phase, indicated by the red square bracket and arrow. Clusters 1 and 2 contain cells with RAM markers that are present during both homeostasis and inflammation and are dominated by M2-like functions in the homeostasis and convalescent phase, marked by blue parentheses and arrows. Clusters 3, 4, and 5 exist only during inflammation and are predominantly characteristic of RecAMs (herein noted by yellow arrows and square brackets). Among them, clusters 3 and 4 are dominated by M1 gene expression (herein annotated by the purple square bracket and arrow). Cluster 5 has a relatively low expression of both M1 and M2 genes. Each cluster has corresponding cellular characteristics that reflect the cell-derived sources and exhibits different functions. RAMs, resident airspace macrophages; RecAMs, recruited airspace macrophages; M, macrophage.

Based on single-cell RNA sequencing, AMs can be identified as five clusters with unique transcriptome characteristics and presumed functions at three different stages (32): physiological homeostasis, acute inflammatory phase, and convalescent phase. The transcripts of clusters 1 and 2 are mainly upregulated in RAMs, while clusters 3, 4, and 5 are predominantly characteristics of RecAMs. Clusters 1 and 2 are dominated by M2 gene expression profiles, while clusters 3 and 4 transcriptomes are dominated by M1 gene expression profiles. RecAM-labeled cells at peak inflammation are dominated by M1 gene expression, while RAM-tagged cells are predominantly expressing the M2 gene at the homeostasis and inflammation phases. The expression of both M1 and M2 genes in cluster 5 is relatively low. RAMs are dominated by M2-like functions in the steady-state phase and convalescent phase, while RecAMs are mainly characterized by M1-like function in the inflammatory phase only (**Figure 3**).

## RSV infection and AM polarization

**Figure 4:**
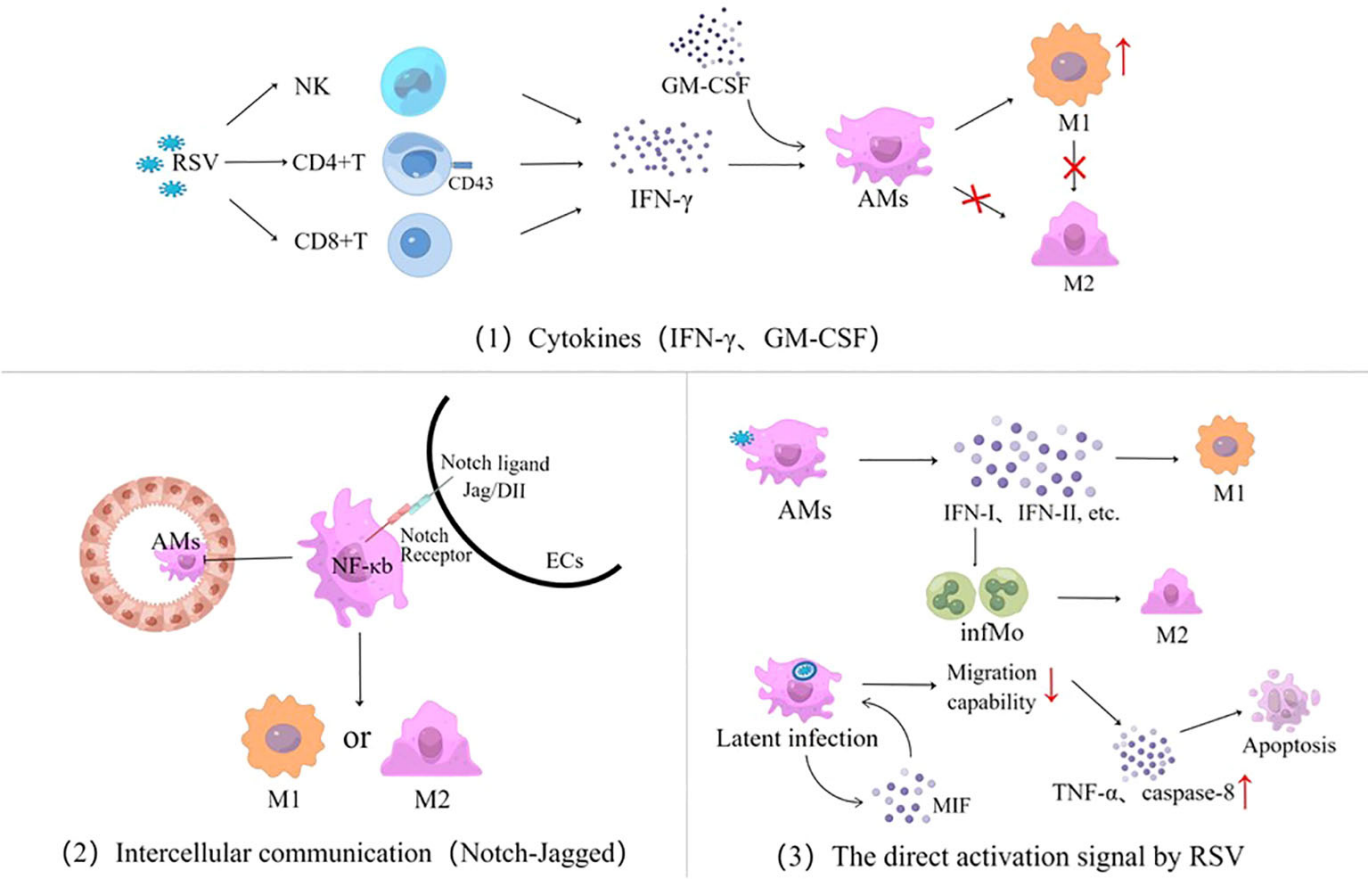
Three signaling pathways for AM polarization activated by RSV infection, cytokines represented by IFN and GM-CSF, intercellular communication using the Notch-Jagged pathway as an example, and the direct activation signal by RSV. RSV, respiratory syncytial virus; NK, natural killer cells; IFN, interferon; GM-CSF, granulocyte–macrophage colony-stimulating factor; AMs, alveolar macrophages; ECs, epithelial cells; M, macrophage; infMo, inflammatory monocytes; MIF, macrophage migration inhibitory factor; TNF-α, tumor necrosis factor-α.

The mechanism by which RSV triggers AM polarization is through promoting a regulatory immune mediator response in three pathways (**Figure 4**): cytokines, intercellular communication signaling (including epithelia–macrophages as well as macrophages–lymphocytes), and RSV invades AMs and directly triggers AM polarization.

### Cytokines

It is well known that IFN-γ is the classic pathway to cause macrophage polarization. RSV infection might stimulate the secretion of IFN-γ from CD8 T cells and NK cells in lung tissues (42–45), which, in turn, regulates inflammatory responses and promotes immunopathology by initiating AM polarization (46). AM polarization activated by IFN-γ is age-related, with significant differences among adults and infants. There is a high level of expression of sialic acid-binding immunoglobulin agglutinin (Siglec-1) ligand CD43 on the membranes of CD4 T cells in adults through antagonizing signals from monocytes and inhibiting the release of IFN-γ by CD4 T cells, thus preventing AMs from polarizing into M1 phenotype. In contrast, due to the lower CD43 expression on CD4 T cell membranes in infants, the IFN-γ secreted by monocyte-mediated CD4 T cells is not affected by Siglec-1 signaling in RSV infection (47). Although infants lack specific memory T cells and their IFN-γ expression is delayed, the role of IFN-γ on AMs gradually dominates as the RSV infection progresses, with the increased CD43 expression being age-related. Therefore, IFN-γ has significant gradual age differences in M1-like polarization effects (11, 48, 49), which is one of the main reasons why the inflammatory response and pathological damage by RSV are different from those of adults (12). GM-CSF also promotes AM polarization in RSV infection, but it plays a secondary role (50).

RSV can also induce the production of pro-inflammatory factors that mediate the expression of macrophage migration inhibitor factor (MIF) through reactive oxygen species, 5-lipoxygenase, cyclooxygenase, and PI3K signaling channels, driving AM polarization to produce TNF-α, monocyte chemoattractant protein-1, and IL-10 (51).

### Intercellular communication

RSV-infected airway ECs might activate AM polarization through intercellular communication such as the Notch–Jagged pathway (24, 52–54). Notch is a ligand–receptor interaction that triggers a highly conserved signaling cascade with a family of four members (Notch 1–4) (55). Notch–Jagged intercellular communication initiates intracellular digestion and modification of the Notch family, by forming a cross-nuclear complex, to initiate AM polarization in coordination with NF-κb signaling and regulates the development of lymphatic lines such as thymus cells, NK cells, and regulatory T cells (Tregs) in the thymus (56, 57). It has been shown that the signal exchange between infected ECs and AMs not only affects the polarization of AMs directly but also further regulates the differentiation and functions of T cell subsets. In addition, ECs can also interact with AMs through the ligand–receptor of CD200 and program death-ligand-1 (24).

### RSV direct activation

AMs can engulf RSV particles directly and recognize viral RNA sequences by PAMPs. *Via* MAVS and RIG-I-like receptors, RSV replication activates AM nuclear transcription to release type I and type II interferons and recruits inflammatory cells (12, 58). RSV infection can maintain inefficient replication within macrophages, forming latent infections (15, 59). By inducing immune cells to express MIF (51), it contributes to weakening the migration of AMs subsequently (5). Through receptor-interacting protein kinase 1 and 3 and mixed-lineage kinase domain-like, RSV upregulates TNF-α and the apoptotic-related gene caspase-8 from the AMs’ autocrine pathway, thereby exacerbating necrotizing apoptosis and lung tissue damage in airway histiocytes (53). RSV invades AMs through inducing the expression of type I IFN to promote the aggregation of inflammatory monocytes (infMo) (12), which can drive M2-like macrophages to express high matrix metalloproteinase-12 and thus exacerbating airway hyperresponsivity (60).

## AM polarization in the different stages of inflammation

To maintain homeostasis, AMs exert mainly immunosuppressive effects by inhibiting the antigen presentation functions of lung dendritic cells or inducing CD4 T cells to be unresponsive (61). It can also secrete a variety of immunomodulatory molecules such as IL-10, TGF-β, NO, and prostaglandin to reduce lung inflammation. Polarized AMs have a dual effect of pro-inflammatory and immune tolerance in the different phases of RSV infection to maintain the intensity of the inflammatory response and the stability of the internal environment and promote tissue repair (34).

### Inflammatory period

Airway ECs and AMs, as the first defense cells of the respiratory tract, can recruit neutrophils through the secretion of molecules to synergistically eliminate pathogens. Damaged lung ECs can induce the loss of the immunosuppressive ligand expression of AMs *via* direct cell–cell contact, which may regulate polarized AMs to M1 phenotype (24). M1 produces pro-inflammatory functions in the acute phase of infection and exhibits a stronger phagocytic activity (62, 63). RSV-mediated AM polarization is mainly through cytokine activation pathways, consisting of IFN-γ, TLR-2, -4, and -9 ligands, lipopolysaccharide, and GM-CSF, manifested as M1-like functions. While inhibiting IL-10 receptor signaling, polarized AMs activate NF-kb nuclear transcription by JAK-STAT1/2 phosphorylation signal to express CD16, to release pro-inflammatory cytokines TNF-α, IL-6, IL-1β, IL-12, and IL-23, and to secrete inducible nitric oxide synthase, which can promote the development of inflammation and upregulate the Th1-like response (64–66). Moreover, in the mitogen-activated protein kinase-dependent pathway, polarized AMs express IL-33 and are capable of activating NF-κB signaling by the production of Th2-related cytokines (13).

AMs are important effector cells to secrete IFN-I, and their secretion levels are age dependent. RSV induces the overexpression of IFN-I in adults but, contrarily, inhibits its production in infants (58). IFN-I inhibits RSV replication by upregulating antiviral gene expression and can also recruit monocytes to differentiate into infMo to exert an antiviral activity (12). Immaturity in the production of IFN-I by infants is one of the molecular bases for their susceptibility to develop severe lung inflammation after an RSV infection. During the acute inflammatory phase, RecAMs are rapidly recruited into the alveoli to participate in the removal of pathogens, promoting inflammation, while RAMs inhibit this inflammation. During the period of inflammation regression, most RecAMs are programmed cell death, while RAMs persist. Within 2 months of infection, the phenotypes and functions of some RecAMs are gradually similar to those of RAMs to supplement the RAMs consumed (67). The increased expressions of IFN-I receptor alpha chain, IFN-induced GTP-binding protein Mx2, 2′–5′-oligoadenylate synthetase 1 (OAS1), OAS2, ribonuclease L, and IFN-induced transmembrane protein 3 in AMs also enhance RSV clearance (68). This phenotype exists in the acute phase of other respiratory virus infections, such as influenza virus (69–72).

### Convalescent period

In the convalescent phase of infection, the AM phenotype is more inclined to M2, which is manifested by the secretion of IL-10 to modulate the Th17-mediated inflammatory response (9), such as upregulating Tregs, inhibiting lung inflammation driven by inflammatory cells (including neutrophils), and promoting tissue repair (68, 73).

AMs are polarized into M2 phenotype mainly under M-CSF stimulation. According to the different cytokine expression profiles, M2 can be divided into three subtypes: M2a, M2b, and M2c. M2a releases a small amount of IL-10, the decoy receptor IL-1RII, and the IL-1 receptor antagonist (IL1ra), predominated by the inflammatory responses of type Th2, which might be associated with airway sensitization. M2b releases the pro-inflammatory factors TNF-α, IL-1, and IL-6 and a large number of IL-10. Dominated by a high level of IL-10, M2b regulates the signals of inactivated immunity and inflammation through inhibiting the proliferation and differentiation of T cells to exert anti-inflammatory and immune-regulating effects. As an anti-inflammator, IL-10 regulates immune and inflammatory signals, including inhibiting the proliferation and differentiation of T cells to exert anti-inflammatory and immune-regulating effects. M2c is activated by autocrine IL-10 and TGF-β, modulating the immune response and assisting in tissue remodeling (65, 74–76).

Thus, during the convalescent phase of lung tissue inflammation, the functions of RAMs and RecAMs gradually switch to the phenotype of different M2 subtypes, promoting tissue repair and pathogen clearance.

### Post-viral AHR

The functional transformation of IMs in the transition from the inflammation period to convalescence is a major intrinsic factor in tissue repair. Early in the convalescent phase of inflammation, M2a is dominated by IL-4 secretion, which, in turn, upregulate the Th2 type immune response leading to AHR, which is associated with wheezing. In the middle and late phases of convalescence, AMs are gradually converted to M2b, mainly secreting IL-10 and TGF-β, regulating the Th17-like immune response negatively, which may promote the production of functional Treg cells, form a positive feedback loop, and inhibit the tolerance of effector T cells to aspiration antigens. IL-10 is mostly secreted by activated IMs by the TLR4/MyD88 pathway. IMs account for about 55% of CD45+ cells that secrete IL-10, compared with less than 5% of CD4 T cells. Activated IMs can impair neutrophil inflammation, mucus production, and the expression of neutrophil-activated cytokines (IL-17, GM-CSF, and TNF-α) in alveoli, negatively regulating the Th2- and Th17-mediated responses (9). In contact with harmless antigens, AMs co-express TGF-β and retinal dehydrogenase 1/2 (77), inducing the production of nTreg cells to maintain immune tolerance (78).

The responses caused by RSV have shown antithesis in immune inflammation and immune tolerance as well as in viral clearance (78). A moderate inflammatory response helps the host defend against pathological harm caused by harmful microorganisms. Decreased immune tolerance can lead to chronic inflammation such as asthma. When infants are re-infected with RSV, the Th1-type immune response might produce IFN-γ, TNF-α, IL-1β, and IL-22 (68, 79), thereby activating CTL and NK cells to clear the virus (10). However, infants are mainly characterized by the Th2- and Th17-like response (80), and the Th2-type immune memory expresses IL-4, IL-5, and IL-13, which down-regulate Th1, leading to reduce the virus clearance rate and increase the inflammation (9, 81). It means that the pathological basis of AHR may be closely related to an excessively unbalanced immune response. During convalescence or RSV re-infection, infants fail to develop airway immune tolerance due to the formation of Th2 immune memory and the down-regulation of Treg cells, which may induce eosinophilic asthma.

In addition, platelets are also involved in the recruitment of immune cells in the regulation of the conversion of AMs’ functions. Stimulated by sCD40L of CD4 T cells, platelets-expressed P-selectin binds to PSGL-1 on the Treg cell membrane to form platelet–Treg aggregates. It is one of the keys to promoting the recruitment of Treg cells to the lungs and releasing anti-inflammatory factors IL-10 and TGF-β. The interaction of platelets with Treg cells is involved in regulating the transcriptional reprogramming of AMs and initiating the polarization of AMs towards anti-inflammatory phenotypes, which effectively relieve lung inflammation (82). At different stages of RSV infection, the phenotypes and functions of AMs change to play a pro-inflammatory and steady-state role, balance and protect the local alveolar microenvironment, and avoid excessive immunopathological damage (59).

## AM-mediated T cell differentiation

Intercellular signaling interactions between airway epithelial cells, AMs, and T lymphocytes may be associated with airway sensitization. RSV might upregulate the expression of Notch signaling protein ligand Dll4 in APCs and lung ECs. The blockade of Dll4 (Notch–Jagged ligand of the signaling pathway) might promote the production of Th2-like cytokines (IL-5 and IL-13), mainly through inducing IL-17A+CD4+T cells to differentiation and IL-17A expression. Thus, it might result in excessive immunopathological damage (57). Upregulated Dll4 promotes T cells to express SET and MYDN domain containing protein 3 through the classic Notch signaling pathway, which contributes to Foxp3 gene methylation and Treg cell differentiation and promotes IL-10 expression (83). Furthermore, RSV promotes the upregulation of Jagged-1 and the downregulation of Jagged-2 in bronchial epithelial cells, which is beneficial to the differentiation of Th2 cells. Besides this, if the expression of Jagged-1 is inhibited, it promotes Th1 and inhibits the differentiation of Th2 cells (54). Thus, the species activity of Notch ligands affects the direction of differentiation of T cells. Whether there are differences in the expression levels of different Notch ligands and whether they are age-related are still unclear.

Polarized AMs affect T cell differentiation in many ways—for example, ultra-fine particles induce AMs to express Jagged-1 and promote allergen-specific T cell differentiation into Th2 and Th17 through the Jagged 1–Notch 4 pathway (84). Lung damage caused by mechanical ventilation upregulates the expression of Notch signal-related proteins and promotes the polarization of AM to M1 phenotype, which, in turn, aggravates airway inflammation (85). Therefore, given the important role of AM polarization and T cell differentiation, experimental evidence is still needed to confirm if RSV infection regulates T cell differentiation through AM polarization, of which it is involved in the later body’s sensitization state. However, after RSV infection, conclusive evidence is needed on how AM polarization affects the imbalance differentiation of T cell associated with the formation of AHR.

## Prospect of AMs as target for the treatment of AHR-related viral infection

Immunomodulatory therapies target AMs that exist in multiple potential sites during a viral infection of the respiratory tract. In the case of rhinovirus infection, AMs can be M1/M2 polarized by GM-CSF/M-CSF or IFN-γ/IL-4 stimulation (86, 87). M1/M2 herein can be likewise classified by their functions and origins rather than dichotomy. In rhinovirus-induced asthma exacerbations, M1-like monocyte-derived macrophages (MDMs) can produce antiviral IFN, while M2-like MDMs significantly enhance the production of Th2-type chemokines (88), where MDMs are commonly classified as RecAMs (89). Furthermore, the inception of rhinovirus-induced AHR may share the analogical pathways with RSV-induced AHR in adaptive immunity—for example, the synergistic interactions between Th2 and Th17 immune responses, in which cytokines (including but not limited to IL-33, IL-13, and IL-17A) are released, mediate eosinophilic and neutrophilic aggregation, jointly inducing AHR (90, 91). After inflammation is controlled, AHR is often characterized by eosinophilic AHR mediated by Th2-like cytokines (IL-5 and IL-13) mediated by immune memory (92). Whether associated with viral infections or the inflammatory cascade, immunomodulatory therapies for AMs will be quite promising and potential.

In the case of homeostasis or M-CSF stimulation, AMs produce anti-inflammatory factors such as IL-10, which result in tissue repair and remodeling similar to those of M2-like functions (93, 94). The current clinical studies of GM-CSF and its receptors are relatively numerous (95)—for instance, the outcomes of severe COVID-19 patients who received a single intravenous dose of mavlimumab to inhibit GM-CSF signaling were relatively better compared with the normal controls (96). However, most of these preclinical research models that inhibit GM-CSF signaling to control inflammation are used in adults and few for infants. Therefore, for RSV infection in infants, a large amount of experimental data is required to prove that GM-CSF and M-CSF signals can target AM polarization. Considering that AMs’ functions in different microenvironments can be reversed, it is necessary to be cautious when using cytokines such as M-CSF to promote the proliferation and polarization of AMs. In homeostasis and convalescence, most AMs are RAMs with M2-like characteristics. Perhaps it is possible to try to obtain RAM-like cells *in vitro* from embryonic liver cells, which have been reported to have similar functions to primary RAMs (97, 98). This may be clinically applied to alveolar lavage therapy (replenishing RAMs) to promote lung repair. In addition, in intercellular signalings, AMs, as APCs, can regulate immune response types that follow through Notch signaling. Combined with Part-6, upregulating Dll4 and Jagged-2 and blocking or downregulating Jagged-1 may inhibit the production of Th2 and Th17-like cytokines and promote Treg cell differentiation.

The desired scenario is to increase virus clearance while maintaining the stability of the lung microenvironment to avoid excessive immune damage. Further studies may be considered from the perspective of IL-10 modulating the adaptive immune response (99, 100). There are currently reports of a hydrogel-based approach to deliver IL-10 to the lung locally without bleeding or other complications (101). This may be a promising clinical treatment strategy.

## Conclusion

In conclusion, RSV infection can affect the polarization of AMs in a variety of ways. At different stages, AMs can regulate the differentiation of T cell by expressing different cytokines to maintain a moderate inflammatory response and homeostasis (102, 103). AMs manifest as M1-like functions, perform pro-inflammatory functions during the early phase of RSV infection, and gradually change to M2. Immunomodulatory therapy targeting AMs is a potential direction for preventing wheezing associated with RSV infection.

## Author contributions

YW and DZ conceptualized the study design. YW, JZ, XW, PY, and DZ wrote the initial drafts of the manuscript. XW and PY revised the text and participated in the modification diagram. All authors contributed to the article and approved the submitted version.

## Funding

This study was funded by the National Natural Science Foundation of China (81670007). This paper was supported by the General Project of Hubei Provincial Health Committee (WJ2021M173).

## Acknowledgments

All figures were made by Figdraw (www.figdraw.com).

## Conflict of interest

The authors declare that the research was conducted in the absence of any commercial or financial relationships that could be construed as a potential conflict of interest.

## Publisher’s note

All claims expressed in this article are solely those of the authors and do not necessarily represent those of their affiliated organizations, or those of the publisher, the editors and the reviewers. Any product that may be evaluated in this article, or claim that may be made by its manufacturer, is not guaranteed or endorsed by the publisher.
